# The application of transperineal ultrasonography combined with shear wave elastography in the evaluation and monitoring of pelvic floor function in the early stage after total hysterectomy

**DOI:** 10.1016/j.clinsp.2025.100656

**Published:** 2025-04-13

**Authors:** Runyan Ji, Jing Wu, Yanqing Xu, Hanzhen Ji

**Affiliations:** Ultrasonography Department, Nantong Third People's Hospital, Affiliated Nantong Hospital 3 of Nantong University, Jiangsu, PR China

**Keywords:** Total hysterectomy, Pelvic floor function, Transperineal pelvic floor ultrasound, Shear Wave Elastography, Puborectalis Muscle

## Abstract

**Background:**

To investigate the combined effect of transperineal ultrasound and Shear Wave Elastography (SWE) in the evaluation and monitoring of pelvic floor functions after total hysterectomy.

**Methods:**

Fifty-two women who planned to undergo total hysterectomy from January 2019 to December 2022 in Nantong Third People's Hospital were selected, and the pelvic floor functions were assessed using transperineal ultrasound combined with SWE. The dynamic alterations in pelvic floor functions at different time points were compared after hysterectomy. The differences in pelvic floor parameters were compared between Transabdominal Total Hysterectomy (TAH) and Laparoscopic Total Hysterectomy (LTH).

**Results:**

The Posterior Urethravesical Angle at rest and Valsalve maneuver (R-PUA, V-PUA), antero-posterior diameter of the hiatus at rest and Valsalve maneuver (R-HAPD, V-HAPD), and elastic modulus of left and right puborectalis at rest (R-L-PRE, R-R-PRE), the Urethral Rotation Angle (URA)were obviously higher at 3 mon after surgery than before surgery and at 1-mon after surgery; the bladder neck-symphyseal distance at rest and Valsalve maneuver (R-BSD,V-BSD), and the difference in elasticity between the left and right puborectalis muscles (L-∆E, R-∆E) were markedly lower at 3 mon than before surgery and at 1 mon after surgery; V-BSD, L-∆E, R-∆E were obviously decreased and the Bladder Neck Descen (BND), URA, V-PUA were obviously increased in the TAH group in comparison with the LTH group.

**Conclusion:**

Pelvic floor functions start to be weak at 3 months after total hysterectomy. TAH causes more significant damage to the structures and functions of the pelvic floor.

## Highlights

• This study explores imaging assessment of pelvic floor function after hysterectomy.
• The TAH can cause more significant damage to the pelvic floor structure and function.
• Transperineal ultrasound and SWE assess pelvic floor function in multiple dimensions.

## Introduction

Pelvic Floor Dysfunction (PFD) is a hot social health issue worldwide, which is not only a physical disease but also leads to a lot of social and psychological problems, which have a lot of negative effects on women's lives. A study has pointed out that pelvic surgery can cause PFD, which is an independent risk factor for the occurrence of PFD.^1^ Total hysterectomy is the most common gynecologic pelvic surgery, which is effective in the treatment of a variety of benign and malignant uterine diseases such as uterine fibroids and endometrial cancer, but inevitably causes PFD, and even the occurrence of PFD at the same time, thus affecting the daily quality of life of patients.^2^^,^^3^

Transperineal Ultrasound (TPUS) can be applied for the anatomical imaging of the pelvic floor, which is obviously superior to physical examination and a variety of imaging examinations, TPUS imaging can reach the same effect as magnetic resonance examination, and it enables real-time dynamic observation of alterations in anatomical structure and position of pelvic floor organ tissue, along with quantification of relevant indicators.^4^

Shear Wave Elastography (SWE) is a reliable quantitative technique that offers insights into the elastic properties of tissues, making it an effective means for evaluating muscles.^5^^,^^6^ SWE is applied to evaluate and monitor the biomechanical characteristics of the muscles in the pelvic floor, and it is a good auxiliary tool for helping TPUS in evaluating the functions of pelvic floor tissue,^7^^,^^8^ thus providing a multi-dimensional and objective basis for evaluating the pelvic floor functions and diagnosing PFD after total hysterectomy.

In previous research, TPUS and SWE were used in combination to investigate the occurrence of PFD after a hysterectomy.^9^ This paper focuses on the alterations in pelvic floor functions at the initial postoperative stage after hysterectomy and the differences in pelvic floor function damage between different surgical methods, which will provide anatomical-functional information support for choosing reasonable prevention and treatment options at the initial postoperative stage in clinic practice.

## Materials and methods

### Study patients

A total of 52 women who would undergo total hysterectomy due to various causes from January 2019 to December 2022 in the hospital were enrolled in this study. The causes of total hysterectomy were multiple uterine fibroids, adenomyosis, cervical dysplasia, endometrial polyps, and refractory dysfunctional uterine bleeding.

The general characteristics of the patients, including age, Body Mass Index (BMI), and pregnancy-labor history, with or without histories of menopause, difficult labor, chronic cough, and constipation, were recorded. The questionnaires were filled out carefully and patiently.

Inclusion criteria: fertile women aged 30‒80 years-old; those with several pregnancies and births of no more than three times, at >10 years after delivery; those with normal understanding and cognitive ability. Exclusion criteria: women with BMI over 28; those presenting with additional conditions such as cough and difficulty in bowel movements that would cause higher abdominal pressure and severe cardiac and pulmonary insufficiency; those who had previously undergone radiotherapy; those with significant pelvic adhesions and receiving rehabilitation treatment of pelvic floor; those who couldn't perform a correct Valsalva maneuver, resulting in poor image clarity; those with diabetes mellitus and a long history of cigarette smoking; those who had given birth to twins or more, and macrosomia.

This prospective study underwent ethical review and obtained approval from the ethics committee of the hospital (EL20210007). The study patients had signed an informed consent form. The present study had a prospective cohort design and followed the STROBE Statement.

### Instruments and methodology

#### Instruments

The transperineal pelvic floor ultrasonography was performed using the Siemens Acuson Sequia ultrasonic diagnostic instrument with a C5–1 convex array probe (at frequencies ranging from 1 to 5 Mhz); the SWE was performed by using a 10L4 linear array probe (at frequencies ranging from 4 to 10 MHz).

#### TPUS examination

**Fig. 1:**
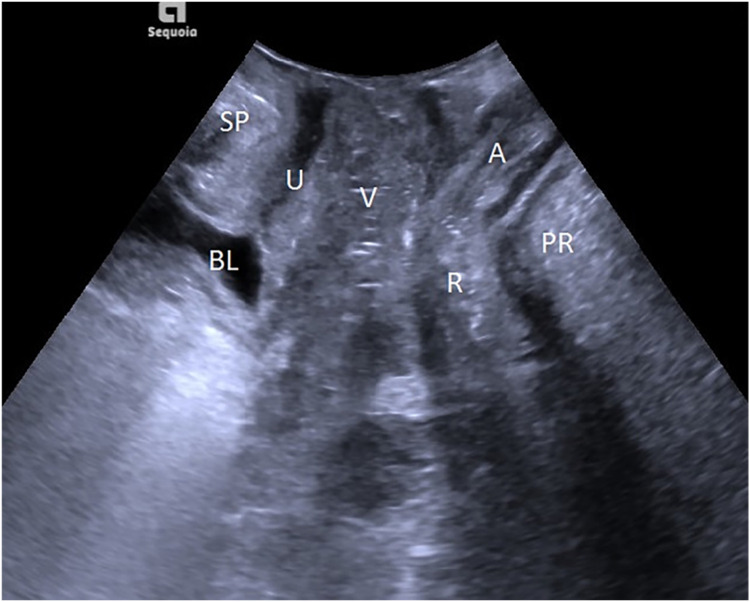
Transperineal two-dimensional pelvic floor median sagittal plane. Bladder (BL), Pubic Symphysis (SP), Urethra (U), Vagina (V), Rectum (R), Anal canal (A), Puborectalis Muscle (PRM).

The patient emptied her bladder with a residual urine volume of less than or equal to 50 mL before the TPUS examination and then took a lithotomy position. The probe was protected with a disposable condom and placed vertically in the center of the perineum to determine a median sagittal plane. From the ventral side to the dorsal side, sequential display of the Symphysis Pubis (SP), urethra, bladder, vagina, anorectal junction, and the central part of the levator plate was performed, as shown in Fig. 1. The horizontal line running across the posterior lower margin of SP was taken as the reference line, and the alterations in the movements of pelvic floor organs and tissues at rest and at maximal Valsalva maneuver, and levator ani contraction were observed, their respective distances from the reference line were measured, and three times of measurement were conducted to determine the lowest point. The states of resting, maximal Valsalva, and levator ani contraction were denoted with the respective letters R, V, and C. Related parameters: Bladder Neck-Symphyseal Distance (BSD), Anorectal Junction-Symphyseal Distance (ASD), with positive values above the line and negative values below the line; Posterior Urethravesical Angle (PUA), and Urethral Obliquity Angle (UOA), as shown in Figure S1. A previous study indicated that the UOA was not statistically significant and thus discarded,^9^ and the Urethral Rotation Angle (URA) derived from UOA was adopted, which is the difference in UOA between resting and maximum Valsalve states; the Bladder Neck Descent (BND) is a difference in BSD between resting and maximum Valsalve states; the Anteroposterior Diameter of the Hiatus (HAPD) is the distance between SP and the medial margin of the levator plate, as shown in Figure S2. Effective Valsalva maneuvers (increasing abdominal pressure to push the pelvic organs to the caudal side) lasted for 6S, and the levator ani contraction lasted for 3S.

#### SWE measurement of the puborectalis muscle

**Fig. 2:**
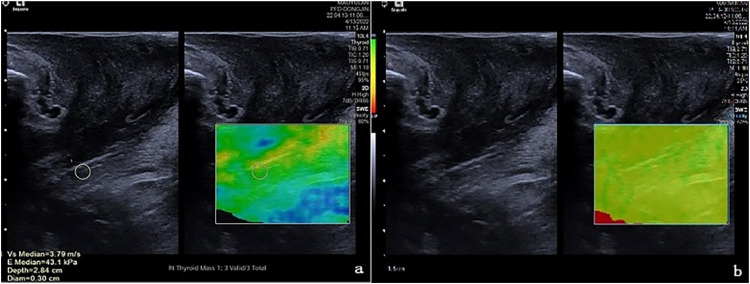
Measurement of PRE at rest. (a) VTIQ velocity diagram; (b) Quality model diagram.

**Fig. 3:**
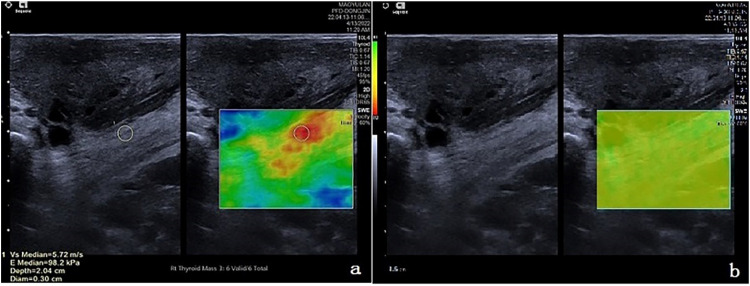
Measurement of PRE during levator ani contraction. (a) VTIQ velocity diagram; (b) Quality model diagram.

The probe was moved to lateral sides, the sound beam obliqued to bilateral puborectalis muscles, and the bilateral slightly hyperechoic puborectalis muscles with slightly hyperechoic were clearly displayed. The 2D-SWE function was activated, and the sampling frame covered the Puborectalis Muscles (PRM) to the maximum extent, and the measurement key was activated during resting and levator ani contraction, respectively, and both the two-dimensional diagram, and the velocity diagram or quality model diagram of Virtual Touch Tissue Imaging Quantification (VTIQ) were displayed sequentially in real-time dynamics. The red-yellow-green-blue colors in the velocity diagram indicated the shear wave velocity from high to low, with a range of 0.5‒10 m/s. The green-yellow-red colors in the quality model diagram indicated the image quality from high to low. When the quality model diagram showed that the green color was evenly distributed, the elasticity of the Puborectalis Muscle (PRM) was measured, the area-of-interest (ROI) (with a size of 3 × 3 mm) was placed in the PRM belly to measure the elastic modulus (Kpa), there were 6‒10 effective measured values, and the median was taken when the Interquartile Range (IQR)/median ratio was less than or equal to 0.3. The sizes and placement locations of ROIs were the same during the repeated measurements. As shown in Fig. 2, Fig. 3.

The TPUS and SWE were performed by 1 specialized sonographer with special training and >15 years of practice.

### Statistical treatment

SPSS 21.0 software (IBM, USA) was applied for data analysis. The test level was α = 0.05 for intergroup comparison. The measured data were described as mean ± standard deviation (*x* ± *s*), and the intergroup comparison was conducted using two independent samples *t*-test; the Mann-Whitney *U* test was used for the measured data without normal distribution, the repeated measurements analysis of variance model was used for comparison among measured data at different timepoints, and Friedman's test was used for the non-normally distributed measured data. The categorical data was described as the number of cases and percentage (%). Graphs were made by Graphpad Prism 7.0.

## Results

### General characteristics

The causes for total hysterectomy were multiple uterine fibroids in 38 patients, adenomyosis in 8 patients, cervical intraepithelial neoplasia in 7 patients, endometrial polyps in 2 patients, refractory dysfunctional uterine bleeding in4 patients, and others in 2 patients. Nine patients were excluded from this study according to the exclusion criteria, and a total of 52 patients who would undergo total hysterectomy were enrolled in the present study. The general characteristics of patients such as age, BMI, parity, and gravidity were presented in Table 1, which indicated no statistically obvious differences in the general characteristics of patients between the TAH group and LTH group (*p* > 0.05).

**Table 1:** Description of general information of patients.

Indicator	TAH group (*n* = 29)	LTH group (*n* = 23)	*t*/*χ^2^*	p
Age	53.483 ± 6.423	52.087 ± 5.575	*t* = 0.824	0.414
BMI (kg/m^2^)	25.178 ± 1.859	24.176 ± 1.767	*t* = 1.972	0.054
Parity	1.414 ± 0.501	1.391 ± 0.499	*t* = 0.161	0.873
Gravidity	2.172 ± 0.966	2.304 ± 0.765	*t* = −0.535	0.595
Menopause/Non-menopause	25/4	20/3	χ^2^ = 0.000	1.000

### Alterations in pelvic floor parameters among different time points before and after surgery

**Fig. 4:**
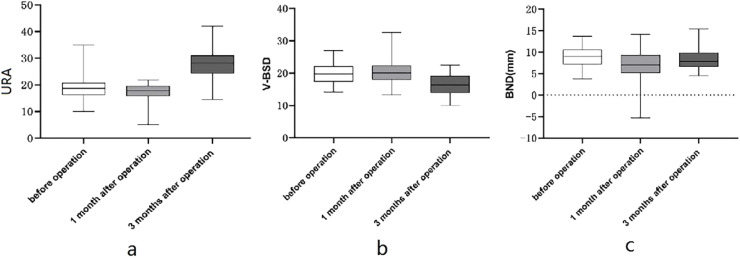
Comparison of conventional pelvic floor ultrasound in patients among 3 timepoints before and after surgery. (a) Alterations in URA among different timepoints before and after surgery; (b) Alterations in V-BSD among different timepoints before and after surgery; (c) Alterations in BND among different timepoints before and after surgery.

**Fig. 5:**
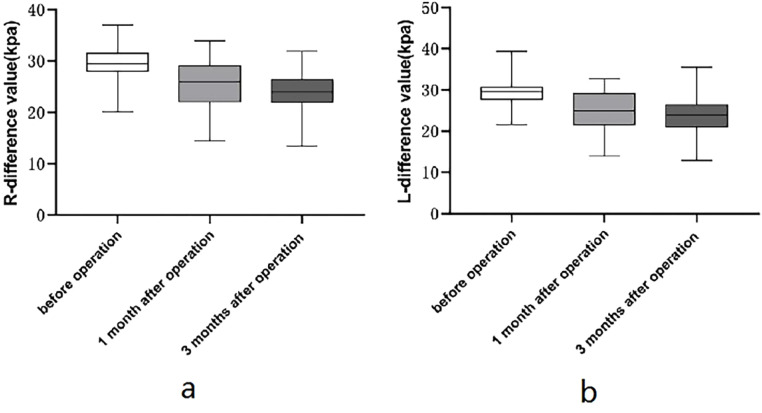
Comparison of SWE of patients among 3 timepoints before and after surgery. (a) Alterations in R-∆E among different timepoints before and after surgery; (b) Alterations in l-∆E among different timepoints before and after surgery.

The results in Table 2 indicated statistically obvious differences in 15 indicators of patients among three time points before and after surgery, including R-BSD, R-PUA, R-ASD, R-HAPD, V-BSD, BND, URA, V-PUA, V-HAPD, R-R-PRE, R-L-PRE, S-R-PRE, S-L-PRE, R-∆E, and L-∆E (*p* < 0.05). In addition, the pairwise comparison results showed that R-PUA, R-HAPD, R-R-PRE, R-L-PRE were obviously higher at 3 mon after surgery in comparison with 1 mon after surgery, and obviously higher at 1 mon after surgery in comparison with before surgery; URA, V-PUA and V-HAPD were obviously higher at 3 mon after surgery in comparison with before surgery and 1 mon after surgery, as shown in Fig. 4; R-BSD, R-ASD, R-∆E, and L-∆E were obviously lower at 3 mon after surgery in comparison with 1 mon after surgery, obviously lower at 1 mon after surgery in comparison with before surgery, see Fig. 5; V-BSD was obviously lower at 3 mon after surgery in comparison with before surgery and 1 mon after surgery, see Fig. 4; BND was obviously lower at 1 mon after surgery in comparison with before surgery and 3 mon after surgery, see Fig. 4; S-R-PRE and S-L-PRE were obviously higher before surgery in comparison with two postoperative time points. There existed no statistically obvious difference in V-ASD among different time points (*p* > 0.05).

**Table 2:** Comparison of conventional pelvic floor ultrasound and SWE in patients among 3 timepoints before and after surgery.

Indicators	Before surgery	1-mon after surgery	3-mon after surgery	*F*	p
R-BSD (mm)	28.435 ± 2.672	26.944 ± 2.765^a^	24.902 ± 2.580^a,^^b^	89.398	0.000
R-PUA (°)	112.046 ± 8.172	116.167 ± 8.644^a^	124.206 ± 11.091^a,^^b^	54.210	0.000
R-ASD (mm)	20.837 ± 2.632	19.837 ± 2.872^a^	17.202 ± 2.910^a,^^b^	73.411	0.000
R-HAPD (mm)	46.327 ± 1.922	47.721 ± 2.389^a^	49.329 ± 2.219^a,^^b^	92.898	0.000
V-BSD (mm)	19.548 ± 2.990	20.171 ± 3.468	16.577 ± 3.308^a,^^b^	37.769	0.000
V-PUA (°)	129.204 ± 8.458	131.408 ± 9.265	138.146 ± 11.529^a,^^b^	28.000	0.000
V-ASD (mm)	14.042 ± 3.172	15.265 ± 12.774	11.690 ± 3.086	2.900	0.060
V-HAPD (mm)	52.000 ± 2.649	51.846 ± 2.728	53.613 ± 3.022^a,^^b^	35.455	0.000
BND (mm)	8.887 ± 2.121	6.773 ± 3.250^a^	8.325 ± 2.471^b^	12.854	0.000
URA (°)	18.292 ± 3.990	17.246 ± 3.412	27.737 ± 5.904^a,^^b^	125.318	0.000
R-R-PRE (kpa)	30.858 ± 3.231	31.706 ± 2.841^a^	33.587 ± 3.475^a,^^b^	55.324	0.000
R-L-PRE (kpa)	31.410 ± 3.118	32.348 ± 3.101^a^	33.758 ± 3.476^a,^^b^	33.894	0.000
S-R-PRE (kpa)	60.277 ± 3.769	57.500 ± 4.786^a^	57.703 ± 4.041^a^	38.177	0.000
S-L-PRE (kpa)	60.694 ± 3.570	57.450 ± 4.536^a^	57.402 ± 3.930^a^	53.571	0.000
R-∆E (kpa)	29.419 ± 3.046	25.794 ± 4.394^a^	24.117 ± 3.922^a,^^b^	75.214	0.000
L-∆E (kpa)	29.285 ± 3.051	25.102 ± 4.555^a^	23.644 ± 4.213^a,^^b^	75.910	0.000

Note: ^a^*p* < 0.05 in comparison with before surgery.

b *p* < 0.05 in comparison with 1 mon after surgery.

### Comparative analysis of various parameters of the pelvic floor between different surgical methods

**Fig. 6:**
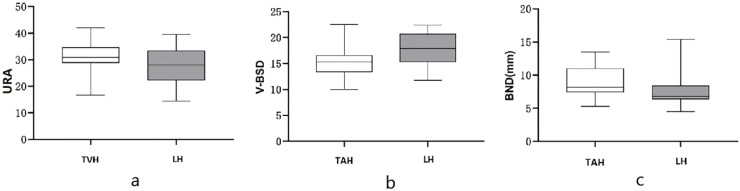
Comparison of conventional pelvic floor ultrasound indicators between two surgery methods. (a) Comparison of URA between two different surgery methods; (b) Comparison of V-BSD between two different surgical methods; (c) Comparison of BND between two different surgical methods.

**Fig. 7:**
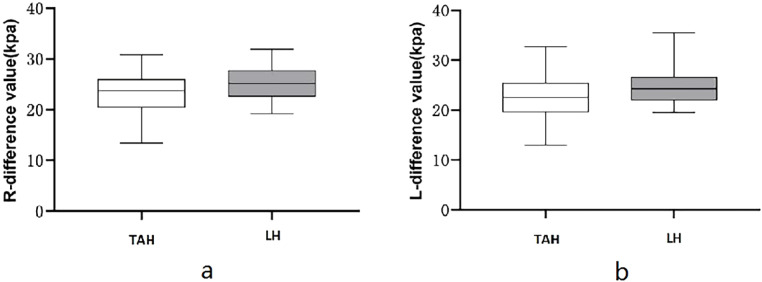
Comparison of SWE after surgery between two surgical methods. (a) Comparison of R-∆E between two different surgical methods; (b) Comparison of L-∆E between two different surgical methods.

The results in Table 3 indicated statistically obvious differences in 6 indicators of patients between the TAH group and the LTH group at 3 months after surgery, including V-BSD, BND, URA, V-PUA, R-∆E, and L-∆E (all *p* < 0.05); V-BSD, R-∆E and L-∆E were obviously lower in the TAH group in comparison with the LTH group, see Figs. 6 and 7; BND, URA, and V-PUA were obviously higher in the TAH group in comparison with the LTH group, see Fig. 6.The differences in the remaining indicators had no statistical significance between statistically significant between two groups (*p* > 0.05).

**Table 3:** Comparison of conventional pelvic floor ultrasound and SWE indicators at 3 mon after surgery between two surgical methods.

Indicators	TAH group (*n* = 29)	LTH group (*n* = 23)	*t*	p
R-BSD (mm)	24.421 ± 2.590	25.509 ± 2.491	−1.530	0.132
R-PUA (°)	124.562 ± 9.373	123.757 ± 13.152	0.258	0.798
R-ASD (mm)	16.721 ± 2.576	17.809 ± 3.239	−1.350	0.183
R-HAPD (mm)	49.786 ± 2.097	48.752 ± 2.280	1.699	0.095
V-BSD (mm)	15.366 ± 3.056	17.843 ± 3.072	−2.897	0.006
V-PUA (°)	142.683 ± 8.343	136.774 ± 11.654	2.050	0.047
V-ASD (mm)	11.338 ± 2.910	12.135 ± 3.305	−0.924	0.360
V-HAPD (mm)	54.031 ± 2.654	53.087 ± 3.419	1.122	0.267
BND (mm)	9.055 ± 2.289	7.665 ± 2.368	2.142	0.037
URA (°)	30.821 ± 5.385	27.326 ± 6.406	2.137	0.037
R-R-PRE (kpa)	33.700 ± 3.537	33.443 ± 3.468	0.262	0.794
R-L-PRE (kpa)	33.872 ± 3.483	33.613 ± 3.541	0.265	0.792
S-R-PRE (kpa)	56.800 ± 4.308	58.842 ± 3.434	−1.853	0.070
S-L-PRE (kpa)	56.241 ± 4.196	58.304 ± 3.182	−1.953	0.056
R-∆E (kpa)	23.100 ± 4.168	25.399 ± 3.236	−2.174	0.034
L-∆E (kpa)	22.369 ± 4.417	24.691 ± 3.571	−2.045	0.046

## Discussion

The uterus is a vital organ in the pelvic floor, and it can exert crucial effects. Therefore, the hysterectomy will inevitably lead to alterations in the structures and functions of the pelvic floor. Based on the three-level theory,^10^ the surgery can disrupt the first level, the apical support, thus breaking the stability of the “hammock”;^11^ based on the holistic theory^12^ transperineal pelvic floor ultrasound, the hysterectomy can create a void in the middle pelvic cavity, thus destroying the perfect integrity of the pelvic floor; the surgery will also lead to the disruption of the sympathetic or parasympathetic nerves distributed in the pelvic floor fascia and ligaments,^13^ and the functions of bladder, bowel, and vagina innervated by these nerves will be destructed to varying degrees.

The comparison of indicators at three different time points before and after total hysterectomy indicates that R-PUA and R-HAPD are obviously higher at 3 mon after surgery in comparison with 1 mon after surgery, and obviously higher at 1 mon after surgery in comparison with before surgery; R-BSD and R-ASD are obviously lower at 3 mon after surgery in comparison with 1 mon after surgery and obviously lower at 1 mon after surgery in comparison with before surgery, so it can be hypothesized that total hysterectomy begins to damage the supportive structures of the pelvic floor gradually. The URA, V-PUA, and V-HAPD are obviously higher at 3 months after surgery in comparison with before surgery and 1 month after surgery; V-BSD was obviously lower at the 3 months after surgery in comparison with before surgery and 1 month after surgery, which indicates that the stability of tissue structures in the anterior and posterior compartments of the pelvic floor are obviously reduced at 3 mon after surgery during Valsalva maneuver, which are in line with the findings of Luo Yannai et al.^14^ Increased mobility of the bladder and urethra at 3 months after surgery indicates that the risk of occurrence of Stress Urinary Incontinence (SUI) will begin to increase, which is consistence with the results of previous studies.^15^^,^^16^ The mobility of the urethra and bladder neck is increased due to the alterations of the spatial positions of the pelvic floor organs after hysterectomy, the amputation of the cardinal ligament and uterosacral ligament during the surgery, and the weakened pelvic floor support and function caused by postoperative damage to pelvic floor tissue and nerves. In addition, the surgery causes damage to the muscles of the pelvic floor and their associated nerves, leading to a reduction in muscle flexibility and adaptability, and enlarged hiatus and HAPD, which aligns with findings from a study by Mou Ruixue et al.^17^ The BND is obviously lower at 1 mon after surgery than before surgery and at 3 months after surgery; it is speculated that most patients do not dare to perform Valsalva maneuver because of pain or fear of incision rupture or poor healing at 1 month after surgery.

A study^18^ suggested that the muscles and fascial ligaments in the pelvic floor can work collaboratively to uphold the stability of the pelvic floor, but under normal conditions, the former provide the main support, and the latter consist of connective tissues and have no sustained tension, thus can only provide temporary support when the muscle damage occurs. As a result, it is of high clinical value to evaluate the performances of the muscles of the pelvic floor. In most of the current literature, the pelvic floor muscle strength assessment indicated that the strength of muscles in the pelvic floor is reduced after hysterectomy,^19^ while there is a lack of standardized electromyographic assessment, which is not conducive to quantitative comparison.^20^ SWE can provide a quantitative evaluation of muscle elasticity, and the results are objective and dependable with a higher degree of reproducibility.^21^^,^^22^ Similar conclusions can be drawn in this study after the SWE evaluation of pelvic floor muscle. R-R-PRE and R-L-PRE are obviously higher at 3 months after surgery in comparison with 1 month after surgery, and obviously higher at 1 month after surgery in comparison with before surgery. It can be hypothesized that the greater the value of elastic modulus is, the greater the tissue hardness is, the lower the elasticity is, and the lower the deformational capacity is,^8^ indicating that the pelvic floor muscle elasticity is damaged after surgery, and the PRE is decreased after surgery, which may be due to the change in PRM components: in the denervated muscle, the atrophy and degeneration of elastic muscle fibers, the fibroblasts without contraction and relaxation functions are increased and deformed, the obviously reduced vascular beds in the muscles accelerate the myocyte degeneration, and the myotome arrangement is disordered with irregular transverse stripes during the muscle cell regeneration, which will result in increased hardness of PRM.^23^ R-∆E and L-∆E are obviously lower at 3 months in comparison with 1 month after surgery, and obviously lower at 1 month after surgery in comparison with before surgery.^9^ The alterations in PRM hardness before and after levator ani contraction can quantify the contractile force of PRM,^22^ indicating that the surgery also affects the contractile forces of the muscles of the pelvic floor; the elastic modulus of the PRM during levator ani contraction at the two postoperative timepoints is lower than that in the preoperative state, which also indicates that the surgery can impair the contraction ability of the muscles of the pelvic floor, and the alterations in the biomechanical characteristics of muscles of the pelvic floor can weaken the supporting function of the pelvic floor as well.

As a result, the women should receive a timely evaluation of their pelvic floor functions after total hysterectomy, similar to the postpartum women. Therefore, the patients should be educated on the importance of receiving early pelvic floor rehabilitation to improve pelvic floor functions promptly, thus enhancing the quality of their life, and SWE evaluation of pelvic floor muscle elasticity after hysterectomy is also worthy of clinical promotion.^24^^,^^25^

Different surgical methods and surgical scopes exert different effects on the structures and functions of the pelvic floor. In this study, the effects of two surgical methods such as TAH and LTH on the structures and functions of the pelvic floor were compared.

This study revealed that TAH had a different effect on the functions of the pelvic floor in comparison with LTH, which was mainly characterized by enhanced bladder neck and urethral mobility, and a weakened PRM contractility. The BND and URA in the TAH group were greater than those in the LTH group, which demonstrated a higher risk of SUI after TAH in comparison with LTH; R-∆E and L-∆E before and after levator ani contraction in the TAH group were lower than those in the LTH group, which indicates that PRM contractility after TAH is lower than that after LTH. It can be concluded that TAH has greater damage to the pelvic floor than TLH. The reason for this is that the bladder needs to be pushed down to the cervical level during TAH, which changes the anatomical relationship and nerve distribution of the organs closely arranged on the pelvic floor and damages the overall structure, anatomical relationship, and physiological state of the pelvic floor. This contradicts the findings of Wang Jie et al., who considered that different surgical methods have similar effects on the pelvic floor.^26^ The reason for this is that the postoperative duration is different. This study explored the differences in the structures and functions of the pelvic floor at 3 mon after surgery between different surgical methods, whereas Wang Jie studied the alterations in the structures and functions of the pelvic floor at 5 years after surgery between different surgical methods. It is hypothesized that the structures and functions of the pelvic floor have been improved after undergoing rehabilitation treatment in the long term, and the damage to the pelvic floor caused by surgical factors will be weakened and gradually counteracted by other factors, these hypotheses need to be confirmed by further studies. Tan Aili et al.^27^ pointed out that there exists no obvious difference in the short-term postoperative effects of surgical paths on the functions of the pelvic floor by studying the incidence of pelvic floor organ prolapse, stress urinary incontinence, and defecation abnormality, and detecting indicators such as pelvic floor muscle fatigue and comprehensive muscle strength after hysterectomy, which is inconsistent with the findings of this study. The reason is that the studied functional indicators of the pelvic floor after surgery are different, thus it is speculated that the ultrasonic parameters of the pelvic floor and the elastic modulus of SWE are more sensitive than other clinical indicators, and this hypothesis also needs to be confirmed by further studies.

## Shortcomings and improvements

This study mainly investigated the functional alterations in the pelvic cavity before and after the total hysterectomy, and there was insufficient research on the middle pelvic cavity because it was found that the vaginal vault was difficult to be displayed when there was no effusion in the rectouterine fossa, and it was impossible to accurately quantitatively assess the vaginal vault prolapse, so it's helpless to give up the study of the middle pelvic cavity. The authors intend to conduct a special study on the condition of the middle pelvic cavity in total hysterectomy in the future after accumulating experience, improving the examination method, and designing a suitable protocol. Fig. 7

Total transvaginal hysterectomy was not included in the subgroup of this study, because the number of cases was too small to reach a minimum sample size; no subgroups of intrafascial and extrafascial TAH were discussed. The authors will collect more cases to improve the follow-up study.

The sample size of this study was relatively small, and the long-term outcome and prognosis of PFD after total hysterectomy were not investigated, and the authors will further conduct a multi-center study on this topic with an expanded sample size in the future.

## Conclusions

Total hysterectomy exerts a negative effect on the pelvic floor support and function at the initial stage, and some structures and functions of the pelvic floor start to weaken at 3 months after surgery; TAH has more significant damage to structures and functions of the pelvic floor than LTH. TPUS can be used for qualitative and quantitative evaluation of the functions of the pelvic floor after hysterectomy, and the SWE can quantitatively assess the biological performances of muscles of the pelvic floor. The combined use of the two examinations can evaluate the functions of the pelvic floor in multiple dimensions, which can provide a more comprehensive and reliable qualitative and quantitative method for effectively preventing, intervening, and delaying the occurrence of PFD after hysterectomy.

## Abbreviations

Pelvic Floor Dysfunction (PFD); Transperineal Ultrasound (TPUS); Shear Wave Elastography (SWE); Body Mass Index (BMI); Symphysis Pubis (SP); Bladder Neck-Symphyseal Distance (BSD); Anorectal junction-Symphyseal Distance (ASD); Posterior Urethravesical Angle (PUA); Urethral Obliquity Angle (UOA); Urethral Rotation Angle (URA); Bladder Neck Descent (BND); Puborectalis Muscles (PRM); Virtual Touch Tissue Imaging Quantification (VTIQ); Puborectalis Muscle (PRM); Area-of-Interest (ROI); Interquartile Range (IQR); Stress Urinary Incontinence (SUI)

## CRediT authorship contribution statement

**Runyan Ji:** Conceptualization, Methodology, Writing – original draft, Project administration. **Jing Wu:** Supervision, Software. **Yanqing Xu:** Data curation, Investigation. **Hanzhen Ji:** Formal analysis, Writing – review & editing.

## Conflicts of interest

The authors declare no conflicts of interest.

## Appendix Supplementary materials

Image, application 1
