# Supplementary material for: The application of transperineal ultrasonography combined with shear wave elastography in the evaluation and monitoring of pelvic floor function in the early stage after total hysterectomy
Source: Clinics (Sao Paulo). 2025 Apr 13;80:100656. doi: 10.1016/j.clinsp.2025.100656 (PMC12017926; doi:10.1016/j.clinsp.2025.100656)

CLINICS-D-24-00735_Supplementary Material

**Figure S1** Schematic diagram of UOA and PUA. (a) Schematic diagram of UOA; (b) Schematic diagram of PUA.


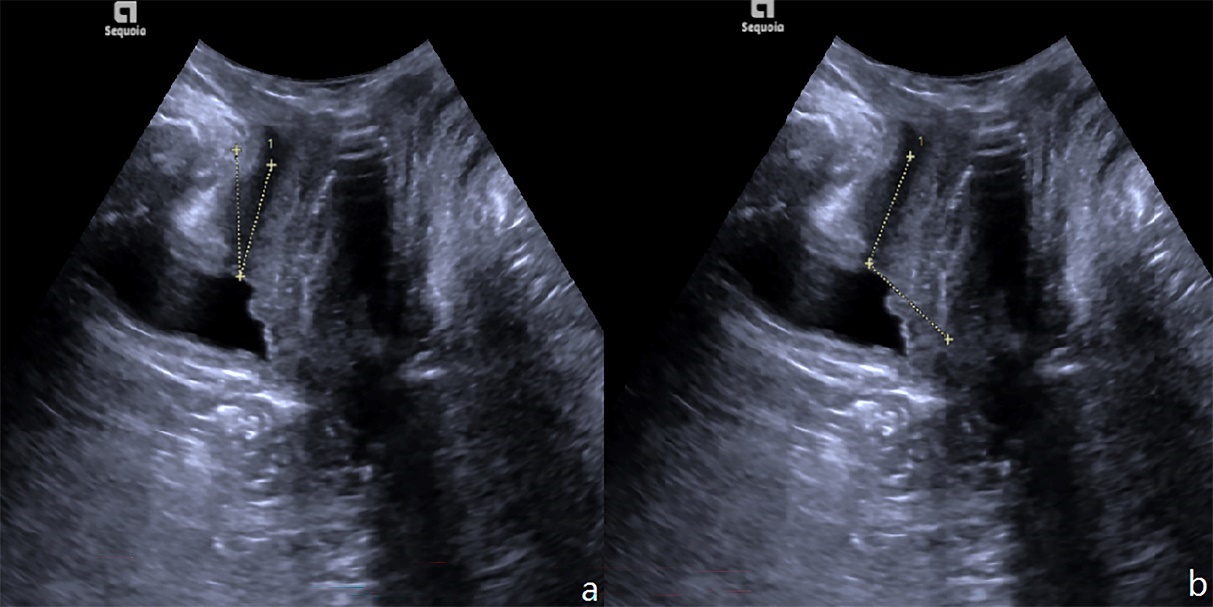


**Figure S2** Schematic diagram of HAPD. (a) At rest; (b) At maximum Valsalve manoeuvre.


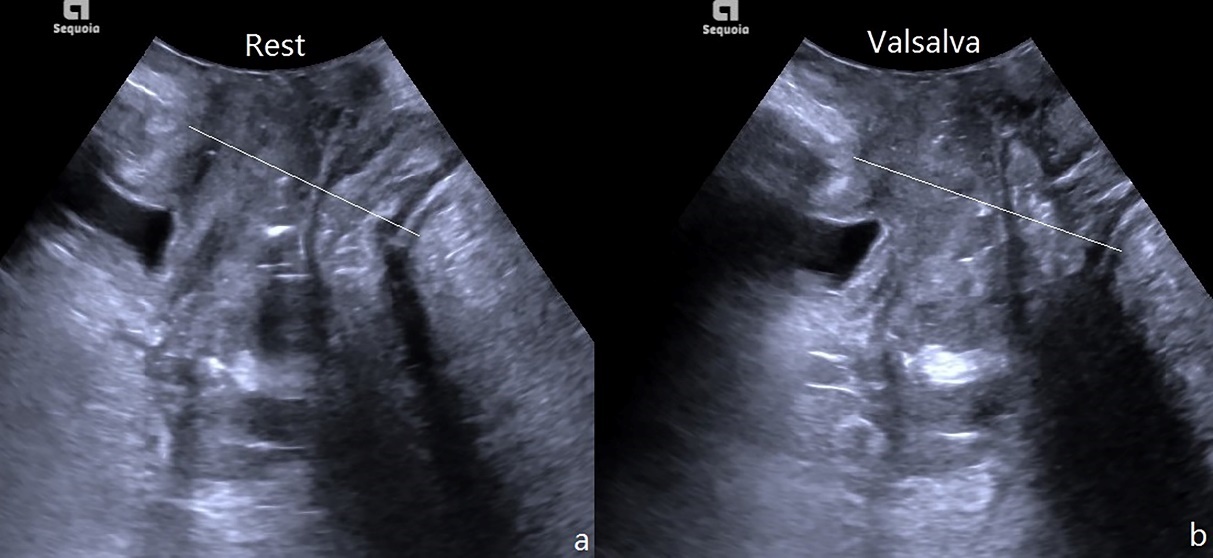

Supplement: Supplementary file 1 [file mmc1.docx]
